# Variations of Bacterial Community Diversity Within the Rhizosphere of Three Phylogenetically Related Perennial Shrub Plant Species Across Environmental Gradients

**DOI:** 10.3389/fmicb.2018.00709

**Published:** 2018-04-18

**Authors:** Xiaofan Na, Tingting Xu, Ming Li, Zhaona Zhou, Shaolan Ma, Jing Wang, Jun He, Bingzhong Jiao, Fei Ma

**Affiliations:** ^1^School of Life Sciences, Ningxia University, Yinchuan, China; ^2^Institute of Environmental Engineering, Ningxia University, Yinchuan, China; ^3^Ningxia (China-Arab) Key Laboratory of Resource Assessment and Environment Regulation in Arid Region, Ningxia University, Yinchuan, China; ^4^College of Resources and Environmental Sciences, Ningxia University, Yinchuan, China

**Keywords:** bacterial community, α diversity, *Caragana* spp., rhizosphere, soil pH

## Abstract

Rhizosphere microbial communities are of great importance to mediate global biogeochemical cycles, plant growth, and fitness. Yet, the processes that drive their assembly remain unclear. The perennial shrubs *Caragana* spp., which is well known for their role in soil and water conservation, provides an ideal system to study the biogeography of rhizosphere microorganism communities within natural ecosystems. In order to detect how bacterial rhizosphere communities vary in terms of community diversity and composition, the rhizosphere bacterial community of three *Caragana* species, *Caragana microphylla* Lam., *C. liouana* Zhao, and *C. korshinskii* Kom., which distributed in arid and semi-arid region of northern China were investigated. Across species, Proteobacteria (61.1%), Actinobacteria (16.0%), Firmicutes (8.6%), Bacteroidetes (3.0%), Acidobacteria (3.5%), Gemmatimonadetes (1.4%), and Cyanobacteria (1.0%) were the most dominant phyla in the rhizosphere of the three *Caragana* species. The relative abundance of Cyanobacteria was significantly higher in rhizosphere of *C. korshinskii* Kom. compared with *C. microphylla* Lam. and *C. liouana* Zhao, while the opposite was found for Gemmatimonadetes in rhizosphere of *C. microphylla* Lam. relative to *C. liouana* Zhao. Stepwise multiple linear regression analysis showed that both diversity and richness of the bacterial rhizosphere communities significantly and positively correlated with soil pH (*p* < 0.01). Distance-based redundancy analysis indicated that soil properties and non-soil parameters detected there accounted for 47.5% of bacterial phylogenetic structure variation (*p* < 0.01) all together. Meanwhile, soil total phosphorus accounted for the greatest proportion of community structure variance (9.7%, *p* < 0.01), followed by electrical conduction (6.5%), altitude (5.8%), soil pH (5.4%), mean annual precipitation (3.6%) and total nitrogen (3.6%, *p* < 0.05 in all cases). Furthermore, partial Mantel test suggested that bacterial rhizosphere community structure significantly correlated with geographical distance, indicating that the less geographical distant sample sites tend to harbor more similar bacterial rhizosphere community. Our results shed new light on the mechanisms of coevolution and interaction between long-lived plants and their rhizosphere bacterial communities across environmental gradients.

## Introduction

Soil is the habitat for a great number of organisms including bacteria, fungi, protozoa, invertebrates, higher plants and animals. These organisms formed complex interaction networks which predominate the functions of the terrestrial ecosystem. Those microbes drive the diverse soil biogeochemical cycles, such as the terrestrial carbon (Bardgett et al., 2008), nitrogen (Stone et al., 2015), and phosphorus cycle (Hilda and Fraga, 1999). Specifically, the term ‘rhizospheric area,’ which was first coined by Hiltner (1904), was used to describe the soil influenced by rhizodeposition of exudates, mucilages, and sloughed cells (Bais et al., 2006). The interactions between microbes in the rhizosphere and plants are of great importance to the element of biogeochemical cycling (Philippot et al., 2009), plant nutrition, health, and productivity (de Vrieze, 2015). Thus, the rhizosphere microbiome is considered as the second genome to plant condition (Berendsen et al., 2012) and serves on a highly evolved external functional environment for plants (Bais et al., 2006; Lakshmanan et al., 2014).

Although previous researchers have detailed the establishment of rhizosphere microbial communities among plant species, questions about the biogeographic pattern and its major drivers of these communities across environmental gradient in natural ecosystems have received far less attention. There are two hypotheses to explain the impact of edaphic and non-edaphic variables on rhizosphere microbes. Firstly, since the composition of any given plant root exudates are dependent on the development stage and physiology status of the plant (Bowsher et al., 2015), the effects of the external factors on the rhizosphere microbial community can be expected to perturbation in plant fitness and metabolism. It has been suggested that the variations of water and nutrients availability, and salinity can modulate the photosynthesis rate and growth of plant, which in turn regulate the composition of rhizosphere bacterial community through inducing changes in exudation pattern (Nessner et al., 2013). Another hypothesis is that the edaphic and non-edaphic variables function as selective pressures that may drive bacterial evolutionary responses directly. For instance, soil pH is often correlated with the observed biogeographical patterns due to the narrow adaptation spectrum of most microorganisms to pH (Fierer and Jackson, 2006). The stress of living in suboptimal pH environments has been shown to have a significant effect on the growth of soil microbial communities (Schnittler and Stephenson, 2000; Bååth, 2010). Under most situations, both the direct and indirect effects of these factors on bacterial rhizosphere community might be performed at the same time. Understanding the mechanisms that maintain and generate the microbial biodiversity in this unique habitat is thus key to predict how ecosystem responses to environmental changes in the future.

The genus *Caragana* is the dominant species in the cold-temperate dry and arid scrublands, deserts, and montane meadows (Zhang et al., 2002) and is well known for its role in sand fixation, soil and water conservation, and as honey resource, fuel, and fodder in China (Ma et al., 2008). Since 1999, this kind of vegetation planting became even more prevalent after the implementation of the “Green for Grain” program initiated by the Chinese government (Jia et al., 2011). So far, more than 100 *Caragana* species have been found in the worldwide which mainly distributed over arid and semi-arid regions of Asia and Europe, whereas at least 60 species of *Caragana* have been found only in China (Yang et al., 2013). Among these *Caragana* species, *Caragana microphylla* Lam., *C. liouana* Zhao, and *C. korshinskii* Kom. are three of the most common species which distribute in the arid and semi-arid regions of the Inner Mongolia plateau. It has been found that *C. microphylla* Lam. mainly distributed in the eastern plateau, *C. liouana* Zhao in the middle, and *C. korshinskii* Kom. in the west of the plateau (Ma et al., 2016). Although the three *Caragana* spp. are usually considered as originated from the same ancestor species (Zhang et al., 2009), they showed substantial divergences in morphological and physiological parameters among them (Ma et al., 2004, 2016) because of the alternative geographical distributions from China’s west to east. As such, this typical divergent evolution processes provided us a unique opportunity to explore the major drivers of the biogeographic pattern of shrub rhizosphere microbial communities.

Here in this study, the diversity and structure of rhizosphere bacterial communities of *Caragana* spp. distributed in the arid and semi-arid regions of northern China were evaluated by using Illumina HiSeq sequencing and multivariate analysis. We hypothesized that (1) the three species of *Caragana*, especially for the *C. korshinskii* Kom., harbor distinct rhizosphere microbiome, (2) edaphic variables and non-soil parameters cooperatively shape the rhizosphere bacterial community structure and (3) soil pH significantly influence the diversity and structure of rhizosphere bacterial communities.

## Materials and Methods

### Site Description

**FIGURE 1:**
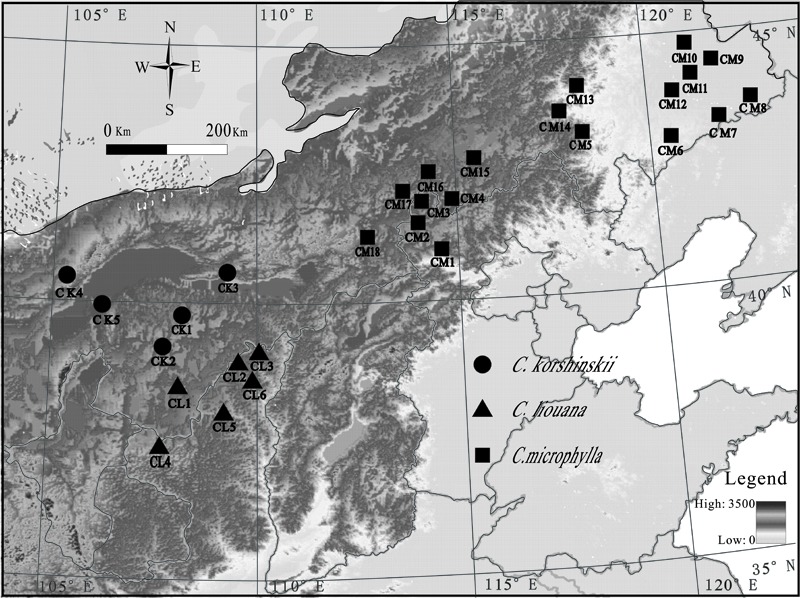
Map of sample locations in northern China. Each symbol represents one sampling site. Samples collected from different sites and species are referred to as *Caragana microphylla* Lam (CM1 to CM16), *C. liouana* Zhao (CL1 to CL6), and *C. korshinskii* Kom (CK1 to CK5). The legend in the figure represents the altitude of the sampling sties.

This study was conducted along over a 2300-km east-west transect across Inner Mongolia in northern China (**Figure 1**). A total of twenty-nine sampling sites, eighteen for *C. microphylla* Lam., six for *C. liouana* Zhao, and five for *C. korshinskii* Kom., were collected from multiple sites through the entire transect in August 2015 (**Figure 1**). And each sampling site was representative of the local natural *Caragana* community. Geographic and climatic information for each sampling site was provided by IWMI online climate summary service portal^1^ and listed in Supplementary Table S1. All the sampling sites located in the region have a temperate continental arid and semi-arid monsoonal climate with mean annual precipitations (MAP) ranged from 103.5 to 417.8 mm. Soil types were dominantly arid, sandy and belonged to Kastanozem soil group according to the Food and Agriculture Organization (FAO) classification system (Wang et al., 2014).

### Sampling and Rhizosphere Soil DNA Extraction

At each site, three independent replicate plots (20 m × 20 m, at least 100 m apart) were chosen. In each plot, both the rhizosphere and bulk soils (soil auger, 5-cm in diameter and 20-cm in length, 20-cm apart from the stem) were collected randomly from at least three individual plants and bulked as a sub-sample, respectively. Then, the three sub-samples were analyzed independently at each sampling site. According to our recent study, the individual plant with similar above-ground biomass was selected to minimize the stand-age effects on community diversity and composition (Na et al., 2017). In order to collect enough rhizosphere soil, three root segments (2 mm or less in diameter and ±5 cm in length) from each individual plant were randomly sampled near the base of the plant. After removing the loosely adhered soil by shaking, the root samples were immediately put into 5 ml tubes with sterilized tweezers and stored in liquid nitrogen immediately.

In the lab, the roots with adhered soils were washed twice with 2.5 ml of sterile 0.9% NaCl solution to collect the rhizosphere soil. The resultant wash was centrifuged at 4°C, 12,000 rpm for 10 min and the deposition was defined as the rhizosphere soil. The rhizosphere soils were then stored at -80°C until further analysis. The rhizosphere soil DNA was extracted from three replicate samples of about 0.1 g wet rhizosphere soil sample using the PowerSoil DNA isolation Kit (Mo Bio, United States) according to the manufacturer’s instruction. DNA purity and concentration was determined by using Nanodrop (ND-2000) and DNA integrity was detected on 1.2% agarose gels.

### Analysis of Soil Properties

The field soils were sieved to 2 mm and small pieces of plant material were removed. Soil pH and electrical conductivity were, respectively, measured using a pH meter and a conductivity meter at a soil/water ratio of 1:5 (w:v) after 2 h in suspension of deionized water (Siqueira et al., 2013). Total organic carbon, total nitrogen and total phosphorus were determined by dichromate oxidation (Chen et al., 2015), Kjeldahl digestion (Chen et al., 2015) and vanadium molybdate yellow colorimetric method (Chen et al., 2015), respectively. The bulk soil properties of these sites are listed in Supplementary Table S2.

### Amplicon Generation and Library Preparation

Partial 16S rDNA-based high-throughput sequencing was used to determine the bacterial diversity and community composition in each sample according to Caporaso et al. (2010). PCR amplification of *16S rRNA* gene V4 region was performed using the specific primers 806R (5′-GGACTACHVGGGTWTCTAAT-3′) and 515F (5′-GTGCCAGCMGCCGCGGTAA-3′) with a 6-bp error-correcting barcode specific to each sample. The V4 region was used because the 806R and 515F primer pair yielded the greatest diversity at bacterial phylum level (Peiffer et al., 2013). All PCR reactions were carried out in 30 μl reactions with 15 μl of Phusion High-Fidelity PCR Master Mix (New England Biolabs, United Kingdom), 0.2 μM of forward and reverse primers, and about 10 ng template DNA. Thermal cycling conditions were an initial denaturation for 1 min at 98 °C, 30 cycles of denaturation at 98°C for 10 s, annealing at 50°C for 30 s, and elongation at 72°C for 60 s, and a final extension at 72°C for 5 min. The PCR products were resolved by 2% agarose gel electrophoresis and samples with bright main strips between 200 and 300 bp were chosen and purified for further analyses.

An equimolar amount of the purified PCR products were submitted to amplicon library preparation by using NEBNext Ultra^TM^ DNA Library Prep Kit for Illumina (NEB, United States) following the manufacturer’s recommendations and index codes were added. Library quality was assessed using a Qubit 2.0 Fluorometer (Thermo Scientific) and Agilent Bioanalyzer 2100 system. Subsequently, high-throughput sequencing was performed on an Illumina HiSeq 2500 platform and 250 bp/300 bp paired-end reads were generated. Raw data from this study have been deposited with the NCBI Sequence Read Archive under accession number SRP132131.

### Sequence Analysis

Paired-end reads from the original DNA fragments were joined using FLASH Software (Magoč and Salzberg, 2011) and then assigned to each sample according to the unique barcodes. The joined pairs were quality filtered with UPARSE software package, and the remaining sequences were clustered into Operational Taxonomic Units (OTUs) at a minimum pair-wise identity of 97% using UPARSE pipeline^2^. The annotate taxonomic information for each representative sequence picked from each OTU was determined using the RDP Classifier (Wang et al., 2007). The OTUs data was then used to calculate the Alpha-diversity (α-diversity) metrics, i.e., rarefaction curves, Shannon index, and Chao 1 diversity, were calculated using QIIME (Caporaso et al., 2010). In order to test the phylogenetic relationships of the OTUs at the phyla level, all the tags in the Core Set (GreenGene database) were aligned using Python Nearest Alignment Space Termination (PyNAST, Version 1.2). Additionally, the Weighted and unWeighted UniFrac distance matrices were calculated by QIIME. These UniFrac distance matrices were used to perform principal coordinate analysis (PCoA) using R (Version 3.3.0) and used to perform UPGMA Clustering using QIIME.

With the help of Barcoded Illumina MiSeq 2500 sequencing, a total of 4,812,737 high quality reads were acquired from replicated samples of 29 sites (Supplementary Table S3). On average, these reads were grouped into 2,568 bacterial OTUs and contained a total of 52,367 taxon tags per sample. The quality control results confirmed the acquired data can be used for further analysis (Supplementary Table S3). Across all samples, a total of 99.6% bacteria and 0.4% archaea were detected from the rhizosphere soil of *C. microphylla* Lam., *C. liouana* Zhao and *C. korshinskii* Kom. (Supplementary Table S4). Taxonomic annotated information of the bacteria indicated that on average 95.4% of taxon tags were assigned to the phylum level and 43.4% were assigned to genus level (Supplementary Table S5).

### Statistical Analysis

One way ANOVA (*Tukey* test) was used to evaluate the differences in bacterial relative abundance or diversity between samples and Pearson correlation analysis was used to evaluate the relationships among bacterial diversity, relative abundance, site characteristics and soil properties by using SPSS (version 22.0). Stepwise regression was performed to linearly analyze relationships between soil properties, non-soil parameters, and the bacterial community diversity using SPSS (version 22.0). In addition, distance-based redundancy analysis (db-RDA) and variance partitioning were performed to investigate relationships between the rhizosphere bacterial community composition, soil properties and non-soil parameters, and using Canoco statistical software (Version 5.0) with default parameter settings according to its tutorial. The correlations between unWeighted or Weighted UniFrac distance matrices and the spatial distance matrices were measured by using partial Mantel test in *R* (*vegan* package).

## Results

### Relationships Between Abiotic Factors and Their Impacts on Bacterial Community Composition

Pearson correlation analyses found that most variables including relative humidity (RH), pH, mean annual temperature (MAT), electric conductivity (EC), total nitrogen content (TN), total phosphorus content (TP), and mean annual precipitation (MAP) were significantly changed with altitude increased (**Table 1**). Meanwhile, **Table 1** showed that soil pH significantly and positively correlated with altitude, PM, and EC, while negatively correlated with RH and MAP. Beside the altitude and pH, MAP also showed significant relationships with TP, RH, and PM. Furthermore, TP significantly correlated with RH, EC as well as PM (**Table 1**).

**Table 1:** Pearson correlation coefficients between soil properties and non-soil parameters across sampling sites.

	Altitude	MAP	MAT	RH	PM	pH	EC	TOC	TN	TP
Altitude	1.00									
MAP	-0.23*	1.00								
MAT	-0.45**	-0.16	1.00							
RH	-0.80**	0.48**	0.08	1.00						
PM	-0.17	-0.54**	0.83**	-0.32**	1.00					
pH	0.50**	-0.52**	0.21	-0.70**	0.41**	1.00				
EC	0.45**	-0.02	-0.24*	-0.35**	-0.09	0.26*	1.00			
TOC	0.21	0.18	-0.28**	-0.02	-0.19	-0.20	0.66**	1.00		
TN	0.32**	0.14	-0.48**	-0.04	-0.39**	-0.06	0.56**	0.65**	1.00	
TP	0.29**	-0.33**	0.06	-0.40**	0.24*	0.32**	0.27*	0.03	0.09	1.00

*TP, total phosphorus content; TN, total nitrogen content; TOC, total organic carbon; EC, electrical conductivity; MAP, mean annual precipitation; MAT, mean annual temperature; RH, relative humidity; PM = Penman–Monteith. ^∗^p < 0.05, ^∗∗^p < 0.01.*

**FIGURE 2:**
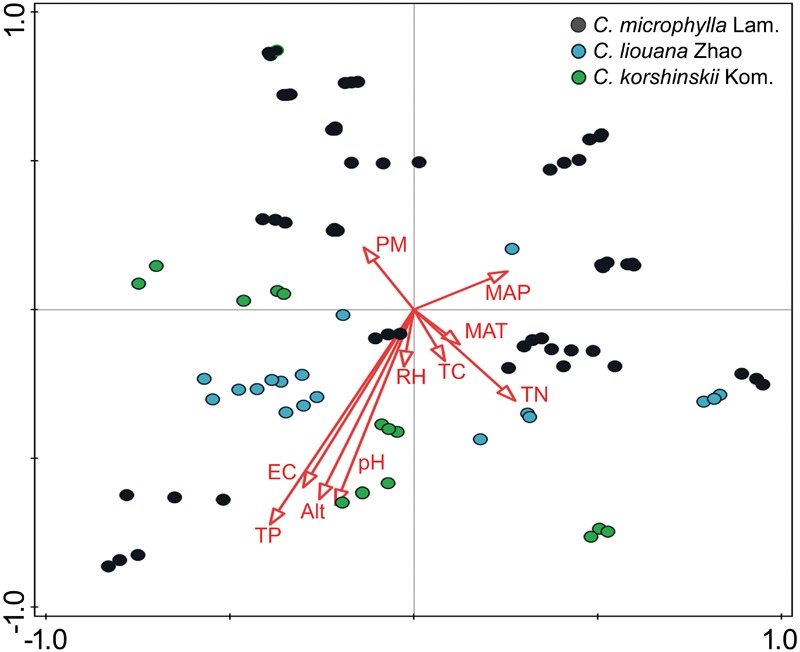
Ordination diagram (samples-environment biplot) of db-RDA depicting environmental drivers of rhizosphere bacterial community composition for *C. microphylla* Lam. (*black circles*), *C. liouana* Zhao (*blue circles*), and *C. korshinskii* Kom. (*green circles*) samples.

The distance-based redundancy analysis (db-RDA) was performed to access to what extent the soil properties and non-soil parameters explain the variance of rhizosphere bacterial community in the three *Caragana* spp. **Table 2** shows that all these factors together explained 47.5% of community variances (*p* < 0.01, **Table 2**), but the soil properties and non-soil parameters together explained 24.7 and 15.5% of community variances, respectively. For each factor, TP, EC, altitude, pH, MAP, and TN contributed significantly to the variance when analyzed individually and TP explained greatest community variances followed by EC, altitude, pH, MAP, and TN (**Figure 2** and **Table 2**). Moreover, the best combination of these factors and its explanation are represented in Supplementary Table S8.

**Table 2:** Results for db-RDA testing effects of soil properties and non-soil parameters on the composition of rhizosphere bacterial communities across all sampling sites.

Variables	% variance explained	*p*-value
All Factors	47.5	0.002
SP	24.7	0.002
EF	15.5	0.002
TP	9.7	0.002
EC	6.5	0.004
Altitude	5.8	0.006
pH	5.4	0.006
MAP	3.6	0.036
TN	3.6	0.034
TC	1.1	0.43
PM	2.1	0.11
MAT	1	0.45
RH	1.3	0.314

*EF, environmental factors; SP, soil properties.*

### Soil pH Significantly Explained Bacterial Community α Diversity and Richness

**FIGURE 3:**
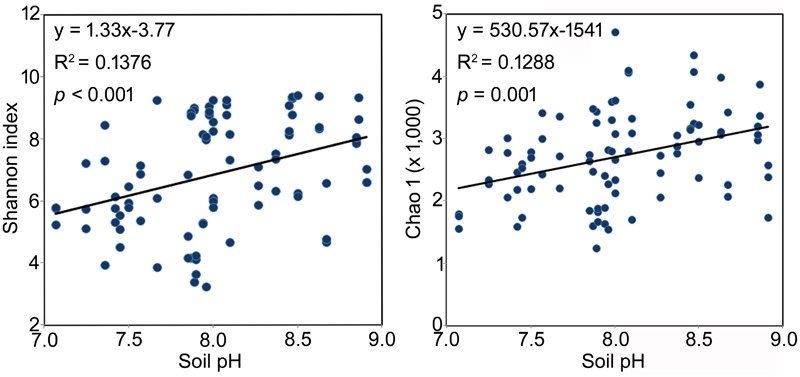
The best model interpreting the changes of bacterial rhizosphere community α diversity of the three *Caragana* spp. Multiple linear regressions were performed using stepwise method. All the soil properties and non-soil parameters were used as independent variables and the Shannon or Chao 1 index were used as dependent variables, respectively.

Across all samples, soil pH, EC, and TP were significantly and positively correlated to the α diversity of the rhizosphere bacterial community, although weakly (Shannon index, *R*^2^ = 0.37, 0.26 and 0.26, respectively, *p* < 0.05 in all cases, **Table 3**). The same significant and weakly positive relationships between soil pH and richness and between TP and richness were detected as well (**Table 3**). Conversely, RH showed significant negative correlations with both α diversity (*R*^2^ = -0.35, *p* < 0.01) and richness (*R*^2^ = -0.33, *p* < 0.01, **Table 3**). Interestingly, the stepwise multiple linear regression model showed that pH was the only significant factor affecting both the α diversity and richness of rhizosphere bacterial community across all soil properties and non-soil variables (Shannon index: *y* = 1.325x-3.768, *p* < 0.001; Chao 1 index: *y* = 530.6x-1541.0, *p* = 0.001, **Figure 3**). The model was not significantly improved if bulk soil TC, TN and TP or all the non-soil parameters were used instead of soil pH alone.

**Table 3:** Pearson correlation analyses testing the relationship between the α diversity index and soil properties and non-soil parameters across all sampling sites.

	Shannon index	Chao 1 index
Altitude	0.207	0.189
MAP	-0.154	-0.177
MAT	0.026	0.032
pH	0.371^∗∗∗^	0.359^∗∗^
EC	0.257^∗^	0.199
TC	-0.058	-0.172
TN	-0.173	-0.169
RH	-0.346^∗∗^	-0.329^∗∗^
PM	0.154	0.173
TP	0.248^∗^	0.278^∗∗^
C/N	0.045	-0.093

*^∗^p < 0.05, ^∗∗^p < 0.01, ^∗∗∗^p < 0.001.*

### The Dominant Bacterial Phyla and Their Relationships With Soil and Non-soil Parameters

**FIGURE 4:**
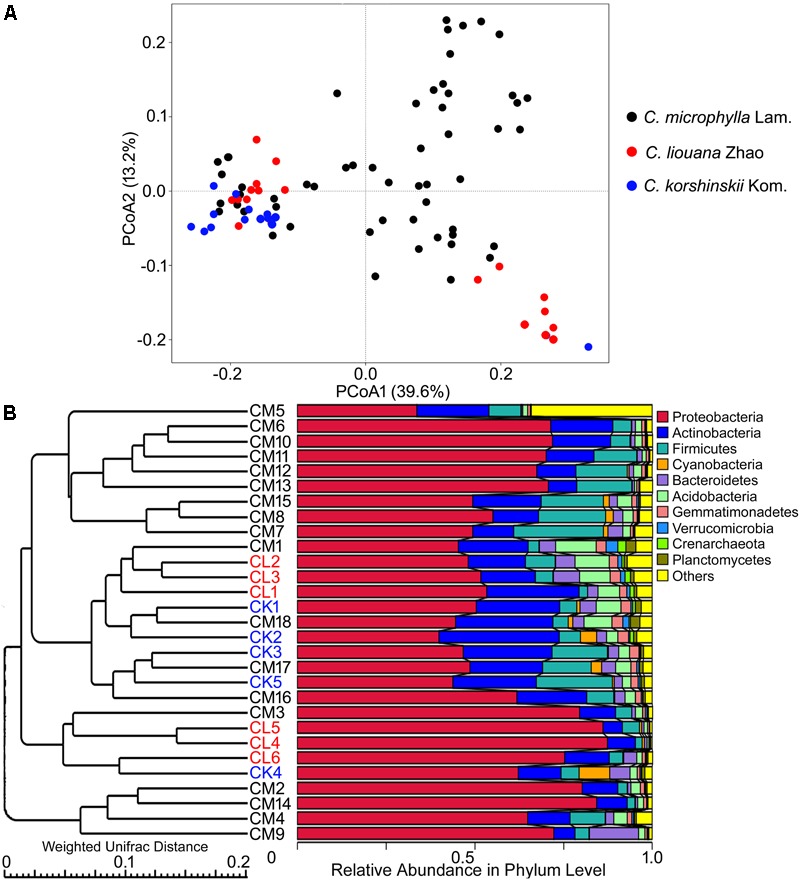
The β-diversity of rhizosphere bacterial communities of three species of *Caragana* sampled from different sites of northern China. **(A)** PCoA profiles for *C. microphylla* Lam (CM), *C. liouana* Zhao (CL), and *C. korshinskii* Kom (CK) displayed using Weighted UniFrac distances. **(B)** UPGMA clustering using Weighted UniFrac distances.

Analysis across all samples of the most dominant phyla in each of OTU rhizosphere of *C. microphylla* Lam., *C. korshinskii* Kom., and *C. liouana* Zhao resulted in the following ten most dominant phyla representing 65.9 to 99.5% of all taxon sequences (**Figure 4** and Supplementary Table S6): Proteobacteria (61.1%); Actinobacteria (16.0%); Firmicutes (8.6%); Acidobacteria (3.5%); Bacteroidetes (3.0%); Gemmatimonadetes (1.4%); Cyanobacteria (1.0%); Planctomycetes (0.7%); Verrucomicrobia (0.5%); and Crenarchaeota (0.3%). One way ANOVA analysis found that the relative abundance of only two phyla showed significant changes between plant species (**Table 4**). The relative abundance of Cyanobacteria was significantly higher in rhizosphere of *C. korshinskii* Kom. compared with *C. microphylla* Lam. and *C. liouana* Zhao, while the relative abundance of Gemmatimonadetes in rhizosphere of *C. liouana* Zhao was more abundance relative to *C. microphylla* Lam. (**Table 4**). At genus level, the ten most dominant genera represented 8.9 to 78.7% of all the taxon sequences (Supplementary Table S7) and were *Pseudomonas, Acinetobacter, Bacillus, Stenotrophomonas, Burkholderia, Paenibacillus, Sphingobacterium, Chitinophaga, Arthrobacter*, and *Chryseobacterium*.

**Table 4:** Differences in relative abundance of dominate bacterial phyla among *C. liouana* Zhao, *C. korshinskii* Kom., and *C. microphylla* Lam. sites.

Phyla	*C. liouana*	*C. korshinskii*	*C. microphylla*
	*Zhao*	Kom.	Lam.
Proteobacteria	60.19 ± 16.29	57.66 ± 20.93	62.23 ± 15.47
Actinobacteria	16.78 ± 7.75	19.91 ± 10.67	14.72 ± 7.06
Firmicutes	6.64 ± 5.00	6.88 ± 6.53	9.74 ± 8.02
Cyanobacteria	0.25 ± 0.35b	3.25 ± 6.91b	0.61 ± 0.91b
Bacteroidetes	3.83 ± 3.66	3.29 ± 2.64	2.70 ± 3.27
Acidobacteria	5.03 ± 3.90	3.02 ± 2.57	3.18 ± 2.71
Gemmatimonadetes	1.86 ± 1.11a	1.80 ± 1.44ab	1.22 ± 0.72b
Chloroflexi	1.37 ± 0.79	1.30 ± 0.81	0.94 ± 0.59
Verrucomicrobia	0.67 ± 0.50	0.37 ± 0.23	0.55 ± 0.81
Crenarchaeota	0.62 ± 0.65	0.45 ± 0.72	0.23 ± 0.63
Planctomycetes	0.67 ± 0.41	0.89 ± 0.70	0.59 ± 0.84

*Data shown are means ± SD. Different lowercases represent significant difference at p < 0.05 level.*

Although the relative abundance of bacterial phyla showed rather high inter-sample variability (Supplementary Table S6), significant correlations between the relative abundance of bacterial phyla and soil and non-soil parameters were detected (**Table 5**). As showed in **Table 5**, RH, altitude, EC, pH and TP were significantly correlated with most of the bacterial phyla. For instance, altitude significantly correlated with the relative abundances of Actinobacteria, Firmicutes, Acidobacteria, Gemmatimonadetes, Verrucomicrobia, and Planctomycetes. RH showed significant relationships with Proteobacteria, Firmicutes, Acidobacteria, Gemmatimonadetes, Verrucomicrobia, Crenarchaeota, and Planctomycetes. And EC have significant relationships with Actinobacteria, Firmicutes, Acidobacteria, Gemmatimonadetes, Verrucomicrobia, Crenarchaeota as well as Planctomycetes (**Table 5**). Conversely, the relative abundances of Actinobacteria, Firmicutes, Acidobacteria, Gemmatimonadetes, Verrucomicrobia, Crenarchaeota, and Planctomycetes were significantly correlated with most of the factors (**Table 5**). Meanwhile, Actinobacteria showed significant relationships with Altitude, MAP, pH, EC, and TP. The relative abundance of Planctomycetes significantly correlated with altitude, RH, pH, EC, and TP. No significant correlation was found between the relative abundance of Bacteroidetes and any variables (**Table 5**).

**Table 5:** Pearson correlation analyses testing the relationship between the relative abundance of dominant rhizosphere bacterial phyla and soil properties and non-soil parameters and across sampling sites.

	Proteobacteria	Actinobacteria	Firmicutes	Cyanobacteria	Bacteroidetes	Acidobacteria	Gemmatimonadetes	Verrucomicrobia	Crenarchaeota	Planctomycetes
Altitude	-0.11	0.28**	-0.32**	0.04	0.17	0.32**	0.42**	0.24*	0.18	0.40**
MAP	0.17	-0.30**	0.05	-0.37**	-0.04	0.12	-0.15	0.09	-0.01	-0.15
MAT	0.06	-0.13	-0.13	0.08	0.14	0.00	-0.08	0.06	0.22*	-0.05
RH	0.26*	-0.40	0.24*	-0.20	-0.10	-0.40**	-0.54**	-0.28**	-0.30**	-0.47**
PM	-0.12	0.08	-0.12	0.23*	0.14	0.11	0.12	0.14	0.27*	0.14
pH	-0.06	0.28**	-0.32*	0.16	0.19	0.21	0.34**	0.16	0.26*	0.32**
EC	-0.15	0.24*	-0.27*	-0.06	0.03	0.49**	0.43**	0.64**	0.39**	0.64**
TC	0.09	-0.13	-0.07	-0.13	-0.06	0.23*	0.04	0.35**	0.19	0.18
TN	0.25*	-0.11	-0.25*	-0.12	-0.11	0.04	-0.02	0.11	-0.05	0.11
TP	-0.19	0.24*	0.05	-0.02	0.05	0.18	0.29**	0.22*	0.20	0.28**

*^∗^p < 0.05, ^∗∗^p < 0.01.*

### Geographic Distance Determined Rhizosphere Bacterial Community Similarity

There is no obvious specie-specific pattern observed among the three *Caragana* spp, although principle coordinate analysis (PCoA) represented clear clustering of different sampling sites (**Figure 4A**). Similarly, the UPGMA clustering analysis also revealed differences in bacterial community composition based on sample sites (**Figure 4B**). In particular, all the samples formed four clear clusters, and two of which included only the rhizosphere samples form *C. microphylla* Lam. The other two clusters showed that the three *Caragana* spp. were not separated as well as the previous two clusters (**Figure 4B**). Furthermore, the geographic distance significantly influenced bacterial community structure across all samples (**Table 6**). Partial Mantel tests detected a significant correlation between spatial distance and Weighted or unWeighted UniFrac distance. In contrast, community distance was not correlated with environmental distance when geographic distance used as a control (**Table 6**). Thus, smaller differences in bacterial rhizosphere community structure are generally observed among pairs of sample sites that are close to one another rather than far from each other.

**Table 6:** The results of Pearson correlation between bacterial community UniFrac distance and soil or non-soil parameter distance for all pairwise samples using partial Mantel test.

	Control factor	Weighted UniFrac distance		Unweighted UniFrac distance	
		ρ	*p*-value	ρ	*p*-value
Geographic distance	Environmental distance	0.2886	0.0005	0.2486	0.0015
Environmental distance	Geographic distance	0.0106	0.3950	-0.1811	0.5328

*p-values are based on 9999 permutations. Environmental distance (Euclidean distance) represents the matrix calculated by the data including pH, EC, TOC, TP, TN, MAP, MAT, RH, PM, and altitude.*

## Discussion

### Soil pH Is a Dominate Predicator for Bacterial Community Diversity but Weak for Community Structure in Rhizosphere of *Caragana* spp. Under Field Conditions

Soil pH is a substantial character for soil quality evaluation that not only affects the growth of plants, but also determines the amount of biogeochemical processes affecting carbon, nitrogen, and phosphorous cycling in soil (Rousk et al., 2010). Previous studies found a positive and significant correlation between soil pH and bacterial community diversity in different conditions and thus suggested that soil pH represents the strongest known predictor of microbial community composition and diversity in surface soils (Fierer and Jackson, 2006; Lauber et al., 2009; Rousk et al., 2010; Bartram et al., 2014). This study further examined the previously found strong correlation between soil pH and bacterial rhizosphere community diversity (**Table 3** and **Figure 3**). The stepwise regression analysis and db-RDA results demonstrated that soil pH is the major determinant of the bacterial rhizosphere community diversity but a poor predictor of the community structure (**Table 2** and **Figure 2**). These results suggest that soil alkalinity might be a vital factor driving the co-evolution process of the plant and its rhizosphere bacterial community, which keeps in lines with previous results about the succession of soil bacteria (Lauber et al., 2009; Rousk et al., 2010; Bartram et al., 2014).

The relationships between soil pH and bacterial rhizosphere community diversity observed here keep in lines with a number of previous studies (Fierer and Jackson, 2006). However, the specific mechanisms underlying this pattern is less known. There are at least three general hypothesis that may explain why soil pH was the best predictor of bacterial rhizosphere community diversity along the transect here. First, soil pH can affect the rhizosphere microbial community directly through microbial toxicity. As has been demonstrated previously, pH directly imposed a physiological constraint on soil bacteria, reducing the net growth, turnover rate and/or regulating interactive/competitive outcomes of individual taxa unable to adapt to soil pH variation (Fierer and Jackson, 2006). The intracellular pH of most microbes is generally within one pH unit of neutral (Madigan et al., 1997), and hence any significant variation in soil pH should impose stress on certain bacteria taxa that cannot adapt. The relationships between pH and the relative abundance of different bacterial phyla could be partially explained the direct effects of pH on taxon sorting (**Table 5**). The second hypothesis is that soil pH indirectly alters the physiological status of the *Caragana* spp., and then the root exudation compositions. The same to the microorganism, extreme pH has been proved to damage the cell structure, inhibit plant growth and mediate the root exudates quantity and quality (Nelson and Mele, 2007; Ibekwe et al., 2010; Zhao et al., 2013). The rhizosphere priming effects of the *Caragana* species in the natural ecosystem might be interfered positively or negatively by the variation of soil pH through regulation of rhizodeposition and/or plant-microbe interaction as well (Kuzyakov, 2002). In addition, a series of site characteristics (altitude, RH, and MAP) and edaphic variables (TP, EC, and C/N) that are often correlated with soil pH (**Table 1**; Brady and Weil, 2007). And it has been confirmed that soil pH can impact the availability of nutrients and heavy metal ion in soil (Harter, 1983; Härdtle et al., 2004). As such, soil pH may act as an integrating factor, which when combined with information on site characteristics and soil conditions regulate the interaction and growth of the microorganisms and plants independently.

In contrast to community diversity, the poor impact of soil pH on bacterial rhizosphere community structure detected in this study is inconsistent with some previous studies using the same method to characterize microbial communities (Lauber et al., 2009). These studies found that pH was also the best predictor of changes of bacterial community composition. This contradiction might suggest that soil pH is not always a universal predictor of bacterial community structure. Depending on the types of habitat and soil, other factors may be more useful than pH for explaining community patterns. For instance, a recent study by Wang et al. (2017) found that soil pH contributing to most bacterial community variations in the typical grassland in the arid and semi-arid region of northern China. Whereas in the desert, desert grassland, and the whole transect, pH weakly or even cannot explain the community similarity. Another study conducted in a typical Tibetan forest ecosystem also found that soil pH determined the diversity but not the structure of soil fungal community (Wang et al., 2015). Although pH has been determined as the best predictor of soil bacterial community structure, it is possible that different soil types and site characteristics would drive the succession pattern of soil bacterial community in distinct habitats, especially for the rhizosphere microenvironment.

The changes in the relative abundances of specific taxonomic groups across the transect are similar to the pH responses observed in bulk soils in other studies (**Table 5**). For example, the relative abundance of Actinobacteria has been shown to increase with pH in soils from many locations across North and South America (Lauber et al., 2009) and in the rhizosphere of two phylogenetically closely related *Caragana* species in northwest China (Na et al., 2017). Also, a positive spearman correlation between pH and relative abundance of Gemmatimonadetes was revealed in a park grass experiment (Zhalnina et al., 2015). However, soil pH demonstrated a positive trend with the relative abundance of Crenarchaeota and Planctomycetes and a negative relationship with Firmicutes in this study, which has not been discerned previously (**Table 5**). The previously detected strong correlation between the relative abundance of Acidobacteria with pH (Dimitriu and Grayston, 2010; Rousk et al., 2010) was not found in our study. These results indicate that the rhizosphere bacterial community might exhibit similar or distinct biogeography patterns when compared with the bulk soil bacterial community.

### Effects of Soil and Non-soil Parameters on Bacterial Rhizosphere Community Except pH

The RH, TP, and EC variables showed significant correlations with alpha diversity index as well (**Table 3**). RH is an important climate variable defined as the ratio of air vapor pressure to the saturated vapor pressure and is used as indicator of the meteorological drought (Farahmand et al., 2015). In opposition to pH, a negative correlation was observed between RH and alpha diversity including Shannon and Chao 1 indices (**Table 3**). It has been suggested that the photosynthetic rate under saturated light increased linearly with increasing air RH (Cui et al., 2009), and hence RH might indirectly influence the quality and quantity of root exudation. In addition, Wang et al. (2014) investigated the relationship between soil N isotopic values and aridity index (the ratio of precipitation to potential evapotranspiration) along a transect across arid and semi-arid regions in China. Their results showed that soil pH decreased with increasing aridity and aridity was the most significant factor influencing soil pH (Wang et al., 2014). As such, another explanation for the negative relationship between RH and α diversity of bacterial rhizosphere community is the indirect effect of RH on soil pH (*r* = -0.70, *p* < 0.01, **Table 1**).

Moreover, our results also found that TP was not only positively correlated with the diversity of the bacterial rhizosphere community (**Table 3**), but was also the most important explanatory variable for community structure (**Table 2**). One hypothesis about these effects is that the growth of the rhizosphere bacteria was directly regulated by phosphorus. It has been widely acknowledged that phosphorus is one of the most important growth limiting edaphic variable for soil microorganisms (Aldén et al., 2001; Ilstedt and Singh, 2005; Chen et al., 2014; Liu et al., 2014). In addition, the essential role of phosphorus for plant growth and the multifunction of soil microorganisms in soil phosphorus solubilization and mineralization (Hilda and Fraga, 1999) might reflect the interactions between plant and rhizosphere bacteria as well.

In the present study, a large amount of the variability in the rhizosphere bacterial community composition remains unexplained, although all of the edaphic variables and site characteristics detected in this study were included in the db-RDA analysis (**Table 2**). These results suggested that the community structure is much more complicated to predict than the community diversity. Consideration of more soil and non-soil variables not measured in this study could improve our ability to predict variations in rhizosphere bacterial community structure across the transect. The bulk soil properties were used to explain the changes in rhizosphere bacterial community composition (**Table 2**), which could have affected our results. The term rhizosphere refers to the distal fraction of rhizospheric area adjacent to rhizoplane, which is still under the roots influence, but without direct contact to the roots (Séguin et al., 2005; Barillot et al., 2012). Thus, it is difficult to acquire enough rhizosphere soils to analyze from the whole of the soils where the three *Caragana* spp. existed. Although the characteristics of the rhizosphere soil were better than that of the bulk soil for the prediction of community variance, a total of 24.7% of community changes can still be explained by the bulk soil variables measured here (*p* < 0.01, **Table 2**). This may not only be because of the rhizosphere bacteria were all recruited from the bulk soil, but also due to the direct effects of bulk soil on root growth.

### Distance-Decay Patterns of Bacterial Rhizosphere Community Structure

Additionally, the partial Mantel test suggested a significant, positive correlation between spatial distance and rhizosphere bacterial community structure dissimilarity (**Table 6**). However, the environmental distance comprised by all the soil and non-soil parameters detected in this study showed no significant relationship with the community structure. This result is inconsistent with that for db-RDA (**Table 2**), possibly because of the differences between the two methods. Generally, the climatic condition is similar across the transect detected in this study. The effects of the variables which changed gradiently from the west to east (such as altitude, MAP, RH, and pH) might be diluted by the consistent parameters (PM, TN, and TOC). Previously, a strong distance-decay relationship between geographic distance and soil bacterial β-diversity was observed in distinct habitats across the arid and semi-arid area of northern China (Wang et al., 2017) and in continental spatial scales (Martiny et al., 2011). And the relative importance of environmental distance vs. geographic distance to bacterial community similarity differed across spatial scales and different habitats (Martiny et al., 2011; Wang et al., 2017). For example, the environmental factors can explain most of the community similarity in alpine grassland, whereas the geographic distance showed a sole effect on community similarity in desert (Wang et al., 2017). Therefore, distinct processes, such as selection, drift, and dispersal limitation, probably have dominant role in shaping bacterial β-diversity pattern in different habitats, respectively. Due to the particularity of the rhizosphere habitat, however, the rhizosphere bacterial biogeographic pattern and the mechanisms driving this pattern need to be further elucidated in the future.

### *Caragana* Species and Bacterial Rhizosphere Community Composition

Recently, Na et al. (2017) investigated the rhizosphere bacterial community composition of *C. jubata* and *C. roborovskii* under natural field conditions in northwest China and found an interspecific clustering of the bacterial rhizosphere communities. This interspecific difference could be partly determined by the genetic differences between the two species (Zhang et al., 2009). In this study, however, only minor interspecific differences in bacterial rhizosphere community were observed between *C. liouana* Zhao and *C. korshinskii* Kom. (**Figure 4**). Among the *C. microphylla* Lam. species, there are two clear clusters in the bacterial rhizosphere communities (**Figure 4**). Similarly, a recent study observed a small fraction of variation in maize rhizosphere community diversity and structure that could be attributed to host genetics in the field conditions (Peiffer et al., 2013). These results suggest that plant species effects on community composition of rhizosphere bacteria are probably not as strong as that of the edaphic and non-edaphic factors within natural ecosystem, in particularly for the phylogenetic related species. Taken together, all of these results indicate a specific biogeographic pattern of the bacterial community within the rhizosphere of *Caragana* spp., which appeared to be determined to a large extent by the interactions among the bulk soil properties, non-soil variables and the plant species, across the arid and semi-arid region of northern China. This biogeographic pattern better explained community diversity using soil pH, but less in community structure.

## Author Contributions

FM designed the research. XN, TX, ML, ZZ, and SM performed the research. FM, TX, JW, JH, and BJ analyzed the data. XN, FM, and TX wrote the manuscript and had primary responsibility for the final content. All authors read and approved the final manuscript.

## Conflict of Interest Statement

The authors declare that the research was conducted in the absence of any commercial or financial relationships that could be construed as a potential conflict of interest.

## Footnotes

1 http://wcatlas.iwmi.org/Default.asp

2 http://drive5.com/uparse/
