# Supplementary material for: Variations of Bacterial Community Diversity Within the Rhizosphere of Three Phylogenetically Related Perennial Shrub Plant Species Across Environmental Gradients
Source: Front Microbiol. 2018 Apr 18;9:709. doi: 10.3389/fmicb.2018.00709 (PMC5915527; doi:10.3389/fmicb.2018.00709)
Supplement: Supplementary file 1 [file Table_1.doc]

**SUPPLEMENTAL DATA**

**TABLE S1** Environmental factors of each sampling site.

| Sample | Species | Latitude | Longitude | Altitude (m) | MAP (mm/y) | MAT (℃/y) | RH (%) | PM (mm/d) |
| --- | --- | --- | --- | --- | --- | --- | --- | --- |
| CL1 | *C. intermedia* Zhao | 38°36'35'' | 108°45'30'' | 1330.0 | 292.2 | 7.3 | 55.8 | 2.9 |
| CL2 | *C. intermedia* Zhao | 39°13'21'' | 110°09'33'' | 1257.0 | 379.9 | 7.2 | 55.4 | 2.9 |
| CL3 | *C. intermedia* Zhao | 39°16'38'' | 110°28'13'' | 1169.0 | 395.0 | 6.9 | 55.2 | 2.8 |
| CL4 | *C. intermedia* Zhao | 37°31'40'' | 108°01'29'' | 1456.0 | 341.7 | 7.3 | 57.5 | 2.7 |
| CL5 | *C. intermedia* Zhao | 38°09'31'' | 109°21'58'' | 1047.0 | 341.5 | 8.4 | 57.0 | 2.9 |
| CL6 | *C. intermedia* Zhao | 38°46'53'' | 110°14'25'' | 1220.0 | 385.8 | 7.4 | 55.8 | 2.9 |
| CK1 | *C. korshinskii* Kom. | 39°50'58'' | 108°41'55'' | 1412.0 | 249.9 | 6.3 | 54.3 | 2.9 |
| CK2 | *C. korshinskii* Kom. | 39°07'2'' | 108°02'44'' | 1400.0 | 223.2 | 6.9 | 54.7 | 2.9 |
| CK3 | *C. korshinskii* Kom. | 40°57'0'' | 110°02'53'' | 1440.0 | 270.1 | 4.7 | 53.9 | 2.8 |
| CK4 | *C. korshinskii* Kom. | 40°46'57'' | 106°32'38'' | 1039.0 | 103.5 | 8.4 | 53.4 | 3.2 |
| CK5 | *C. korshinskii* Kom. | 40°08'44'' | 106°53'25'' | 1180.0 | 121.8 | 7.7 | 54.0 | 3.1 |
| CM1 | *C. microphylla* Lam. | 40°52'43'' | 114°44'56'' | 970.0 | 352.3 | 8.5 | 58.1 | 3.0 |
| CM2 | *C. microphylla* Lam. | 41°37'52'' | 114°18'41'' | 1341.0 | 336.4 | 3.9 | 57.1 | 2.7 |
| CM3 | *C. microphylla* Lam. | 41°56'13'' | 114°44'08'' | 1472.0 | 341.9 | 2.5 | 57.3 | 2.6 |
| CM4 | *C. microphylla* Lam. | 41°53'23'' | 115°13'31'' | 1611.0 | 369.6 | 2.9 | 57.5 | 2.6 |
| CM5 | *C. microphylla* Lam. | 42°59'49'' | 119°09'32'' | 626.0 | 326.8 | 6.2 | 61.6 | 2.8 |
| CM6 | *C. microphylla* Lam. | 42°37'18'' | 120°53'19'' | 450.0 | 369.8 | 6.9 | 62.5 | 2.8 |
| CM7 | *C. microphylla* Lam. | 42°45'40'' | 121°55'7'' | 227.4 | 401.6 | 7.1 | 62.8 | 2.8 |
| CM8 | *C. microphylla* Lam. | 43°03'59'' | 122°17'09'' | 234.0 | 417.8 | 6.6 | 63.1 | 2.7 |
| CM9 | *C. microphylla* Lam. | 44°02'24'' | 121°53'48'' | 1178.0 | 331.4 | 6.7 | 63.1 | 2.7 |
| CM10 | *C. microphylla* Lam. | 44°19'18'' | 120°55'38'' | 285.1 | 329.6 | 6.5 | 63.0 | 2.7 |
| CM11 | *C. microphylla* Lam. | 43°53'40'' | 121°15'41'' | 236.9 | 328.2 | 7.0 | 63.0 | 2.8 |
| CM12 | *C. microphylla* Lam. | 43°38'17'' | 120°55'06'' | 287.5 | 327.1 | 7.1 | 62.9 | 2.8 |
| CM13 | *C. microphylla* Lam. | 43°44'7'' | 119°5'44'' | 759.5 | 338.4 | 4.5 | 61.7 | 2.6 |
| CM14 | *C. microphylla* Lam. | 43°22'39'' | 118°31'32'' | 728.0 | 322.2 | 5.7 | 61.4 | 2.8 |
| CM15 | *C. microphylla* Lam. | 42°40'54'' | 116°00'27'' | 1326.0 | 338.1 | 1.4 | 58.6 | 2.5 |
| CM16 | *C. microphylla* Lam. | 42°28'52'' | 114°51'40'' | 1266.0 | 309.8 | 2.5 | 57.8 | 2.6 |
| CM17 | *C. microphylla* Lam. | 42°04'33'' | 114°20'12'' | 1419.0 | 325.4 | 2.5 | 57.0 | 2.6 |
| CM18 | *C. microphylla* Lam. | 41°16'28'' | 112°55'33'' | 1789.0 | 344.6 | 2.2 | 55.1 | 2.5 |

Notes: MAP, mean annual precipitation; MAT, mean annual temperature; RH, relative humidity; PM, Penman-Montieth. Samples collected from different sites and plants are referred to as *C. intermedia* Zhao (CL1, CL2, CL3, CL4, CL5 and CL6), *C. korshinskii* Kom. (CK1, CK2, CK3, CK4 and CK5) and *C. microphylla* Lam. (CM1, CM2, CM3, CM4, CM5, CM6, CM7, CM8, CM9, CM10, CM11, CM12, CM13, CM14, CM15, CM16, CM17 and CM18).

**TABLE S2** Soil properties of the bulk soil collected from each sampling site.

| Sample | Species | TOC (%) | TN (%) | TP (%) | pH | EC (µS /cm) |
| --- | --- | --- | --- | --- | --- | --- |
| CI1 | *C. intermedia* | 0.33 ± 0.06 | 0.009 ± 0.000 | 0.056 ± 0.003 | 8.5 ± 0.2 | 20.3 ± 1.5 |
| CI2 | *C. intermedia* | 0.60 ± 0.17 | 0.004 ± 0.000 | 0.032 ± 0.029 | 7.9 ± 0.3 | 35.3 ± 3.8 |
| CI3 | *C. intermedia* | 0.11 ± 0.06 | 0.004 ± 0.000 | 0.019 ± 0.002 | 8.9 ± 0.4 | 33.7 ± 2.1 |
| CI4 | *C. intermedia* | 0.52 ± 0.11 | 0.005 ± 0.000 | 0.033 ± 0.004 | 8.5 ± 0.2 | 46.6 ± 3.7 |
| CI5 | *C. intermedia* | 0.14 ± 0.02 | 0.006 ± 0.000 | 0.032 ± 0.002 | 8.0 ± 0.4 | 17.4 ± 1.3 |
| CI6 | *C. intermedia* | 0.43 ± 0.03 | 0.006 ± 0.000 | 0.044 ± 0.006 | 8.1 ± 0.5 | 34.4 ± 2.7 |
| CK1 | *C. korshinskii Kom* | 0.29 ± 0.01 | 0.013 ± 0.000 | 0.038 ± 0.005 | 8.7 ± 0.7 | 38.9 ± 2.0 |
| CK2 | *C. korshinskii Kom* | 0.60 ± 0.15 | 0.012 ± 0.000 | 0.039 ± 0.003 | 8.3 ± 0.2 | 41.5 ± 3.4 |
| CK3 | *C. korshinskii Kom* | 0.27 ± 0.03 | 0.004 ± 0.000 | 0.051 ± 0.003 | 8.6 ± 0.2 | 32.0 ± 2.4 |
| CK4 | *C. korshinskii Kom* | 0.22 ± 0.10 | 0.004 ± 0.000 | 0.031 ± 0.002 | 8.9 ± 0.5 | 31.1 ± 1.8 |
| CK5 | *C. korshinskii Kom* | 0.22 ± 0.10 | 0.011 ± 0.000 | 0.279 ± 0.087 | 8.9 ± 0.7 | 37.8 ± 2.6 |
| CM1 | *C. microphylla Lam* | 0.38 ± 0.08 | 0.011 ± 0.000 | 0.032 ± 0.003 | 8.3 ± 0.5 | 45.8 ± 3.1 |
| CM2 | *C. microphylla Lam* | 1.48 ± 0.21 | 0.107 ± 0.003 | 0.040 ± 0.001 | 7.9 ± 0.4 | 50.7 ± 7.3 |
| CM3 | *C. microphylla Lam* | 1.66 ± 0.01 | 0.166 ± 0.002 | 0.043 ± 0.001 | 7.9 ± 0.2 | 47.2 ± 1.5 |
| CM4 | *C. microphylla Lam* | 1.70 ± 0.04 | 0.119 ± 0.002 | 0.040 ± 0.003 | 7.9 ± 0.2 | 75.0 ± 3.9 |
| CM5 | *C. microphylla Lam* | 0.12 ± 0.02 | 0.006 ± 0.000 | 0.008 ± 0.001 | 7.4 ± 0.3 | 20.3 ± 3.6 |
| CM6 | *C. microphylla Lam* | 0.31 ± 0.02 | 0.020 ± 0.000 | 0.011 ± 0.000 | 8.0 ± 0.6 | 28.0 ± 0.7 |
| CM7 | *C. microphylla Lam* | 0.25 ± 0.02 | 0.004 ± 0.000 | 0.014 ± 0.001 | 7.7 ± 0.4 | 13.0 ± 0.8 |
| CM8 | *C. microphylla Lam* | 0.31 ± 0.04 | 0.006 ± 0.000 | 0.011 ± 0.000 | 7.3 ± 0.2 | 12.8 ± 1.0 |
| CM9 | *C. microphylla Lam* | 0.52 ± 0.03 | 0.036 ± 0.000 | 0.020 ± 0.003 | 7.9 ± 0.2 | 25.8 ± 1.6 |
| CM10 | *C. microphylla Lam* | 0.57 ± 0.04 | 0.064 ± 0.002 | 0.018 ± 0.001 | 8.4 ± 0.2 | 54.7 ± 6.3 |
| CM11 | *C. microphylla Lam* | 0.24 ± 0.04 | 0.013 ± 0.000 | 0.010 ± 0.000 | 7.4 ± 0.3 | 18.4 ± 0.9 |
| CM12 | *C. microphylla Lam* | 0.54 ± 0.07 | 0.006 ± 0.000 | 0.019 ± 0.001 | 7.5 ± 0.6 | 21.6 ± 1.2 |
| CM13 | *C. microphylla Lam* | 1.34 ± 0.26 | 0.003 ± 0.000 | 0.030 ± 0.001 | 7.1 ± 0.3 | 33.7 ± 3.8 |
| CM14 | *C. microphylla Lam* | 0.22 ± 0.03 | 0.024 ± 0.000 | 0.010 ± 0.000 | 7.5 ± 0.7 | 16.4 ± 0.8 |
| CM15 | *C. microphylla Lam* | 0.42 ± 0.02 | 0.007 ± 0.000 | 0.014 ± 0.000 | 7.6 ± 0.3 | 22.9 ± 1.2 |
| CM16 | *C. microphylla Lam* | 0.20 ± 0.12 | 0.001 ± 0.000 | 0.013 ± 0.001 | 8.5 ± 0.5 | 36.0 ± 2.5 |
| CM17 | *C. microphylla Lam* | 0.62 ± 0.01 | 0.008 ± 0.000 | 0.026 ± 0.001 | 8.0 ± 0.5 | 32.4 ± 2.8 |
| CM18 | *C. microphylla Lam* | 0.24 ± 0.01 | 0.089 ± 0.000 | 0.077 ± 0.003 | 8.0 ± 0.1 | 75.5 ± 5.7 |

Notes: TP, total phosphorus content; TN, total nitrogen content; TOC, total organic carbon; EC, electrical conductivity.

**TABLE S3** Statistics of quality control of the Barcoded Illumina HiSeq 2500 sequencing results.

| Sample | Mean raw reads | Mean high quality reads | Mean taxon tag | Mean read length | Mean OUT number | Mean GC content (%) | Mean effective (%) |
| --- | --- | --- | --- | --- | --- | --- | --- |
| CI1 | 58406 | 53616 | 49084 | 258 | 3112 | 56.0 | 89.2 |
| CI2 | 52610 | 50160 | 46710 | 256 | 3276 | 55.0 | 92.3 |
| CI3 | 59792 | 58893 | 57422 | 253 | 1474 | 52.9 | 96.8 |
| CI4 | 65820 | 63775 | 59167 | 253 | 2988 | 55.5 | 93.1 |
| CI5 | 52805 | 51829 | 50456 | 253 | 1643 | 53.2 | 96.4 |
| CI6 | 48006 | 47017 | 45022 | 253 | 2068 | 53.7 | 95.3 |
| CK1 | 58502 | 57318 | 52116 | 253 | 3363 | 55.8 | 93.7 |
| CK2 | 48972 | 47872 | 44909 | 253 | 3012 | 56.3 | 95.3 |
| CK3 | 59748 | 57782 | 53924 | 254 | 3693 | 55.3 | 93.9 |
| CK4 | 88583 | 85337 | 80698 | 253 | 2634 | 54.0 | 93.3 |
| CK5 | 37516 | 36584 | 34073 | 253 | 2133 | 55.6 | 94.3 |
| CM1 | 52057 | 49921 | 46019 | 254 | 3565 | 56.0 | 93.0 |
| CM2 | 57820 | 56744 | 54645 | 253 | 2459 | 56.1 | 96.1 |
| CM3 | 68570 | 66948 | 64136 | 253 | 2458 | 54.4 | 95.1 |
| CM4 | 52855 | 51680 | 49363 | 253 | 2590 | 54.9 | 95.4 |
| CM5 | 57630 | 56571 | 55058 | 252 | 1976 | 51.3 | 96.6 |
| CM6 | 54495 | 53177 | 50549 | 253 | 2329 | 54.7 | 94.7 |
| CM7 | 56706 | 55510 | 52914 | 253 | 2287 | 54.0 | 95.3 |
| CM8 | 64066 | 62244 | 58902 | 253 | 2855 | 54.5 | 94.1 |
| CM9 | 55653 | 54555 | 52287 | 253 | 2131 | 54.4 | 95.4 |
| CM10 | 54532 | 53443 | 51134 | 253 | 2280 | 54.3 | 95.4 |
| CM11 | 58623 | 57241 | 54578 | 253 | 1960 | 54.5 | 94.7 |
| CM12 | 49642 | 48461 | 46173 | 253 | 2124 | 54.5 | 94.8 |
| CM13 | 51197 | 50233 | 48405 | 253 | 1715 | 53.3 | 95.7 |
| CM14 | 57628 | 56419 | 54002 | 253 | 2228 | 54.8 | 95.1 |
| CM15 | 61455 | 60160 | 56694 | 253 | 2866 | 55.0 | 94.8 |
| CM16 | 52703 | 51673 | 48623 | 253 | 2834 | 54.9 | 94.7 |
| CM17 | 53425 | 52188 | 48504 | 253 | 3006 | 55.3 | 94.0 |
| CM18 | 58183 | 56897 | 53080 | 253 | 3415 | 56.1 | 95.0 |
| Average | 56828 | 55319 | 52367 | 253 | 2568 | 54.7 | 94.6 |

**TABLE S4** The percentage (± SD; N = 3) of bacteria and archaea in different species of *Caragana.*

| Sample | Bacteria (%) | Archaea (%) | Sample | Bacteria (%) | Archaea (%) |
| --- | --- | --- | --- | --- | --- |
| CI1 | 98.8 | 1.2 | CM5 | 100.0 | 0.0 |
| CI2 | 99.4 | 0.6 | CM6 | 99.9 | 0.1 |
| CI3 | 100.0 | 0.0 | CM7 | 99.9 | 0.1 |
| CI4 | 99.7 | 0.3 | CM8 | 99.9 | 0.1 |
| CI5 | 100.0 | 0.0 | CM9 | 100.0 | 0.0 |
| CI6 | 99.8 | 0.1 | CM10 | 99.9 | 0.1 |
| CK1 | 99.3 | 0.7 | CM11 | 100.0 | 0.0 |
| CK2 | 98.9 | 1.1 | CM12 | 99.8 | 0.2 |
| CK3 | 98.4 | 1.6 | CM13 | 99.9 | 0.1 |
| CK4 | 99.9 | 0.1 | CM14 | 100.0 | 0.0 |
| CK5 | 99.7 | 0.3 | CM15 | 99.9 | 0.1 |
| CM1 | 97.8 | 2.2 | CM16 | 99.8 | 0.2 |
| CM2 | 99.9 | 0.1 | CM17 | 99.9 | 0.1 |
| CM3 | 99.9 | 0.1 | CM18 | 99.6 | 0.4 |
| CM4 | 99.7 | 0.3 | Average | 99.6 | 0.4 |

**TABLE S5** The mean percentage abundance (± SD; N = 3) of taxon tags in bacterial rhizosphere communities of different species of *Caragana* at different levels of taxonomic classification.

| Sample | Kindom | Phyla | Class | Order | Family | Genus | Species |
| --- | --- | --- | --- | --- | --- | --- | --- |
| CI1 | 95.1 ± 0.7 | 94.4 ± 0.7 | 92.4 ± 0.6 | 85.9 ± 0.1 | 65.5 ± 1.5 | 28.4 ± 0.4 | 7.2 ± 0.8 |
| CI2 | 96.1 ±0.4 | 92.0 ± 1.2 | 90.1 ± 1.0 | 84.2 ± 1.3 | 63.8 ± 5.1 | 33.8 ± 4.5 | 10.3 ± 3.3 |
| CI3 | 96.2 ±0.6 | 94.5 ± 1.0 | 92.5 ± 1.5 | 85.2 ± 4.0 | 64.9 ± 7.7 | 35.5 ± 7.9 | 11.1 ± 6.5 |
| CI4 | 96.9 ± 0.3 | 96.7 ± 0.3 | 96.0 ± 0.5 | 91.7 ± 1.6 | 77.7 ± 2.3 | 45.2 ± 4.7 | 11.0 ± 2.8 |
| CI5 | 99.1 ± 0.3 | 98.9 ± 0.4 | 98.6 ± 0.5 | 97.5 ± 0.8 | 92.7 ± 1.6 | 73.6 ± 5.4 | 22.4 ± 4.8 |
| CI6 | 98.2 ± 1.2 | 98.0 ± 1.3 | 97.6 ± 1.5 | 95.4 ± 2.7 | 84.2 ± 12.8 | 62.0 ± 19.0 | 34.7 ± 24.2 |
| CK1 | 95.1 ± 1.1 | 94.8 ± 1.0 | 93.4 ± 0.9 | 87.2 ± 1.3 | 68.2 ± 3.4 | 29.9 ± 2.5 | 6.0 ± 0.2 |
| CK2 | 96.2 ± 0.3 | 94.9 ± 0.3 | 93.5 ± 0.2 | 87.6 ± 2.1 | 65.3 ± 6.1 | 32.5 ± 1.9 | 9.9 ± 0.7 |
| CK3 | 99.2 ± 0.2 | 99.1 ± 0.2 | 99.0 ± 0.2 | 98.0 ± 0.4 | 93.6 ± 1.7 | 82.1 ± 2.9 | 43.1 ± 5.0 |
| CK4 | 97.4 ± 1.7 | 96.9 ± 2.0 | 96.4 ± 2.4 | 93.8 ± 4.2 | 70.8 ± 24.5 | 50.1 ± 27.8 | 25.3 ± 30.7 |
| CK5 | 96.4 ± 0.4 | 96.2 ± 0.4 | 95.5 ± 0.4 | 91.2 ± 0.1 | 73.8 ± 0.2 | 39.1 ± 0.4 | 7.9 ± 0.6 |
| CM1 | 94.9 ± 0.5 | 94.2 ±0.6 | 91.9 ± 0.9 | 84.7 ± 1.5 | 60.7 ± 0.9 | 28.4 ± 0.6 | 6.8 ± 0.8 |
| CM2 | 98.4 ± 0.3 | 98.1 ±0.3 | 97.5 ± 0.5 | 95.1 ± 1.0 | 87.6 ± 2.9 | 17.2 ± 6.0 | 6.0 ± 2.5 |
| CM3 | 98.5 ± 0.1 | 98.4 ±0.2 | 97.9 ± 0.2 | 95.9 ± 0.6 | 88.5 ± 1.4 | 39.0 ± 4.3 | 16.8 ± 2.7 |
| CM4 | 97.8 ± 0.3 | 94.8 ± 2.7 | 94.1 ± 2.8 | 90.9 ± 3.4 | 81.0 ± 5.5 | 34.5 ± 18.4 | 5.8 ± 2.3 |
| CM5 | 98.9 ± 0.2 | 65.9 ± 21.3 | 65.5 ± 21.4 | 64.0 ± 21.6 | 59.0 ± 21.4 | 27.7 ± 6.7 | 13.9 ± 7.9 |
| CM6 | 98.0 ± 0.1 | 97.8 ± 0.1 | 97.2 ± 0.2 | 94.9 ± 0.5 | 86.1 ± 1.4 | 45.7 ± 1.4 | 23.1 ± 3.6 |
| CM7 | 98.0 ± 0.3 | 94.1 ±1.3 | 93.4 ± 1.4 | 91.6 ± 1.7 | 83.2 ± 3.9 | 56.7 ± 6.2 | 30.5 ± 3.0 |
| CM8 | 97.6 ± 0.4 | 95.3 ± 0.9 | 94.4 ± 1.2 | 91.7 ± 1.7 | 80.0 ± 3.1 | 52.1 ± 3.7 | 26.1 ±10.6 |
| CM9 | 98.5 ± 0.2 | 98.3 ± 0.3 | 97.9 ± 0.5 | 96.3 ± 0.9 | 90.4 ± 2.0 | 41.9 ± 9.0 | 23.8 ± 5.6 |
| CM10 | 98.2 ± 0.2 | 98.0 ± 0.2 | 97.7 ± 0.2 | 95.6 ± 0.5 | 88.5 ± 1.6 | 47.6 ± 8.3 | 27.2 ± 10.4 |
| CM11 | 98.4 ± 0.3 | 98.3 ± 0.3 | 98.1 ± 0.4 | 96.7 ± 0.5 | 91.1 ± 0.9 | 58.0 ± 4.2 | 28.6 ± 2.3 |
| CM12 | 98.1 ± 0.3 | 97.7 ± 0.2 | 97.3 ± 0.3 | 95.2 ± 0.7 | 87.3 ± 2.0 | 44.4 ± 18.3 | 26.2 ± 19.9 |
| CM13 | 98.8 ± 0.2 | 95.9 ± 1.9 | 95.6 ± 1.9 | 94.4 ± 2.1 | 90.0 ± 2.6 | 64.5 ± 3.2 | 49.7 ± 3.4 |
| CM14 | 98.5 ± 0.1 | 98.2 ± 0.1 | 97.7 ± 0.2 | 95.6 ± 0.4 | 89.0 ± 1.4 | 19.2 ± 0.9 | 5.4 ± 0.9 |
| CM15 | 97.3 ± 0.3 | 95.7 ± 0.5 | 94.5 ± 0.3 | 91.1 ± 0.7 | 77.5 ± 3.9 | 48.5 ± 5.5 | 17.7 ± 9.0 |
| CM16 | 97.5 ± 0.3 | 97.1 ± 0.4 | 96.4 ± 0.4 | 93.0 ± 0.3 | 78.0 ± 2.6 | 49.5 ± 6.1 | 6.9 ± 1.3 |
| CM17 | 96.5 ± 0.6 | 96.1 ± 0.9 | 95.2 ± 1.0 | 91.1 ± 1.6 | 72.1 ± 3.5 | 40.1 ± 2.8 | 8.0 ± 0.6 |
| CM18 | 96.1 ± 0.5 | 95.8 ± 0.5 | 94.7 ± 0.5 | 87.3 ± 1.0 | 64.3 ± 0.5 | 29.0 ± 0.7 | 5.6 ± 0.9 |
| Average | 97.5 ± 1.3 | 95.4 ± 6.8 | 94.5 ± 6.9 | 91.1 ± 7.5 | 78.5 ± 12.3 | 43.4 ± 16.9 | 17.9 ± 14.2 |

**TABLE S6** Relative abundance (%) of the dominant bacterial phyla in rhizosphere soils of different species of *Caragana*.

| Sample | Proteobacteria | Actinobacteria | Firmicutes | Bacteroidetes | Acidobacteria | Gemmatimonadetes | Planctomycetes | Cyanobacteria | Verrucomicrobia | Crenarchaeota | Others |
| --- | --- | --- | --- | --- | --- | --- | --- | --- | --- | --- | --- |
| CI1 | 53.3 ± 3.4 | 26.1 ± 1.1 | 2.4 ± 0.4 | 2.9 ± 1.1 | 6.1 ± 1.8 | 2.0 ± 0.1 | 0.8 ± 0.3 | 0.04 ± 0.01 | 0.9 ± 0.1 | 1.14 ± 0.09 | 4.3 ± 0.3 |
| CI2 | 48.2 ± 2.6 | 16.1 ± 2.0 | 8.4 ± 3.6 | 5.4 ± 4.0 | 9.7 ± 4.6 | 2.4 ± 0.8 | 0.9 ± 0.5 | 0.15 ± 0.06 | 1.1 ± 0.5 | 0.58 ± 0.38 | 7.1 ± 1.2 |
| CI3 | 51.7 ± 2.5 | 15.4 ± 2.1 | 4.9 ± 1.1 | 7.4 ± 5.5 | 8.7 ± 1.0 | 2.9 ± 1.2 | 1.1 ± 0.2 | 0.12 ± 0.02 | 1.2 ± 0.4 | 1.55 ± 0.78 | 5.1 ± 1.4 |
| CI4 | 46.7 ± 3.3 | 25.0 ± 2.4 | 15.6 ± 4.7 | 3.0 ± 0.6 | 3.0 ± 0.3 | 2.6 ± 0.7 | 0.7 ± 0.2 | 0.22 ± 0.07 | 0.4 ± 0.1 | 0.26 ± 0.05 | 2.5 ± 0.6 |
| CI5 | 86.0 ± 3.2 | 5.5 ± 2.0 | 4.8 ± 1.1 | 0.6 ± 0.2 | 1.0 ± 0.4 | 0.5 ± 0.2 | 0.1 ± 0.0 | 0.72 ± 0.78 | 0.1 ± 0.0 | 0.02 ± 0.01 | 0.6 ± 0.2 |
| CI6 | 75.2 ± 14.1 | 12.6 ± 6.3 | 3.8 ± 1.2 | 3.7 ± 4.9 | 1.6 ± 0.8 | 0.8 ± 0.3 | 0.4 ± 0.3 | 0.26 ± 0.09 | 0.3 ± 0.2 | 0.15 ± 0.06 | 1.2 ± 0.6 |
| CK1 | 50.5 ± 4.2 | 23.5 ± 1.1 | 4.8 ± 1.7 | 4.6 ± 0.7 | 6.9 ± 2.5 | 2.8 ± 0.5 | 1.6 ± 0.7 | 0.88 ± 0.84 | 0.6 ± 0.2 | 0.69 ± 0.54 | 3.1 ± 0.8 |
| CK2 | 39.9 ± 5.2 | 33.8 ± 2.2 | 6.0 ± 2.3 | 2.8 ± 0.9 | 3.1 ± 0.5 | 3.1 ± 2.0 | 0.9 ± 0.6 | 4.57 ± 4.44 | 0.4 ± 0.2 | 1.05 ± 1.43 | 4.4 ± 0.2 |
| CK3 | 87.4 ± 2.4 | 7.9 ± 0.9 | 1.9 ± 0.8 | 0.7 ± 0.3 | 0.5 ± 0.1 | 0.3 ± 0.1 | 0.1 ± 0.0 | 0.64 ± 0.69 | 0.1 ± 0.0 | 0.02 ± 0.02 | 0.5 ± 0.1 |
| CK4 | 62.2 ± 26.4 | 12.1 ± 9.9 | 5.1 ± 2.0 | 5.7 ± 4.6 | 2.0 ± 1.4 | 0.8 ± 0.5 | 0.9 ± 0.7 | 8.72 ± 14.68 | 0.4 ± 0.2 | 0.13 ± 0.10 | 2.1 ± 1.4 |
| CK5 | 43.8 ± 0.6  45.4 ± 4.0 | 23.5 ± 2.3  19.7 ± 2.1 | 21.6 ± 0.0  3.0 ± 0.8 | 2.3 ± 0.6  4.5 ± 1.0 | 2.4 ± 0.1  11.5 ± 1.7 | 2.0 ± 0.1  2.8 ± 0.5 | 0.9 ± 0.2  2.7 ± 0.1 | 0.51 ± 0.11  0.11 ± 0.05 | 0.5 ± 0.2  3.3 ± 1.2 | 0.32 ± 0.03  2.22 ± 1.96 | 2.2 ± 0.5  4.7 ± 0.5 |
| CM1 |
| CM2 | 80.3 ± 6.1 | 10.1 ± 4.1 | 2.7 ± 1.1 | 0.8 ± 0.2 | 2.8 ± 0.8 | 1.0 ± 0.1 | 0.4 ± 0.2 | 0.07 ± 0.02 | 0.3 ± 0.1 | 0.07 ± 0.06 | 1.4 ± 0.2 |
| CM3 | 79.5 ± 3.0 | 10.2 ± 1.9 | 4.5 ± 1.0 | 1.0 ± 0.1 | 2.0 ± 0.2 | 0.9 ± 0.2 | 0.3 ± 0.0 | 0.03 ± 0.01 | 0.4 ± 0.2 | 0.13 ± 0.07 | 1.1 ± 0.2 |
| CM4 | 64.9 ± 14.3 | 11.9 ± 2.8 | 9.9 ± 10.1 | 2.3 ± 2.0 | 3.8 ± 0.9 | 1.3 ± 0.3 | 0.5 ± 0.2 | 0.10 ±0.07 | 0.4 ± 0.2 | 0.28 ± 0.24 | 4.5 ± 2.9 |
| CM5 | 33.6 ± 10.0 | 20.4 ± 18.1 | 9.0 ± 5.4 | 0.4 ± 0.1 | 1.4 ± 0.2 | 0.7 ± 0.3 | 0.1 ± 0.0 | 0.06 ± 0.01 | 0.1 ± 0.0 | 0.02 ± 0.01 | 34.1 ± 21.4 |
| CM6 | 71.4 ± 2.9 | 17.5 ± 1.5 | 5.2 ± 1.6 | 1.1 ± 0.1 | 1.7 ± 0.5 | 0.9 ± 0.3 | 0.2 ± 0.1 | 0.14 ± 0.07 | 0.2 ± 0.0 | 0.12 ± 0.06 | 1.5 ± 0.3 |
| CM7 | 49.4 ± 4.5 | 11.6 ± 6.3 | 25.3 ± 3.9 | 4.2 ± 0.7 | 2.2 ± 0.9 | 0.8 ± 0.2 | 0.2 ± 0.0 | 1.20 ± 0.82 | 0.2 ± 0.1 | 0.05 ± 0.05 | 4.9 ± 1.3 |
| CM8 | 55.0 ± 5.9 | 12.9 ± 2.4 | 18.8 ± 9.3 | 2.7 ± 0.8 | 3.0 ± 0.6 | 1.1 ± 0.3 | 0.2 ± 0.1 | 2.18 ± 0.94 | 0.4 ± 0.1 | 0.08 ± 0.08 | 3.6 ± 1.4 |
| CM9 | 72.2 ± 5.0 | 6.0 ± 1.1 | 3.9 ± 0.8 | 13.9 ± 5.6 | 1.7 ± 0.5 | 0.6 ± 0.2 | 0.2 ± 0.1 | 0.09 ± 0.04 | 0.3 ± 0.0 | 0.01 ± 0.01 | 1.0 ± 0.3 |
| CM10 | 71.9 ± 5.1 | 16.4 ± 6.1 | 5.5 ± 1.9 | 1.1 ± 0.1 | 2.0 ± 0.5 | 0.9 ± 0.2 | 0.3 ± 0.2 | 0.23 ± 0.09 | 0.3 ± 0.1 | 0.08 ± 0.04 | 1.3 ± 0.2 |
| CM11 | 70.1 ± 2.0 | 13.4 ± 0.9 | 12.1 ± 2.0 | 1.4 ± 0.5 | 1.2 ± 0.1 | 0.7 ± 0.1 | 0.1 ± 0.0 | 0.08 ± 0.03 | 0.1 ± 0.0 | 0.04 ± 0.01 | 0.7 ± 0.1 |
| CM12 | 67.4 ± 12.0 | 11.1 ± 2.6 | 14.5 ± 8.5 | 1.4 ± 0.1 | 2.2 ± 0.3 | 1.0 ± 0.3 | 0.2 ± 0.1 | 0.44 ± 0.23 | 0.2 ± 0.1 | 0.18 ± 0.22 | 1.4 ± 0.3 |
| CM13 | 70.7 ± 16.3 | 8.0 ± 1.0 | 15.5 ± 14.3 | 0.6 ± 0.1 | 0.7 ± 0.1 | 0.6 ± 0.3 | 0.1 ± 0.0 | 0.08 ± 0.03 | 0.1 ± 0.0 | 0.08 ± 0.08 | 3.5 ± 1.7 |
| CM14 | 84.3 ± 1.7 | 8.7 ± 0.9 | 2.1 ± 0.2 | 0.6 ± 0.1 | 1.7 ± 0.5 | 0.9 ± 0.2 | 0.2 ± 0.0 | 0.10 ± 0.01 | 0.2 ± 0.0 | 0.00 ± 0.01 | 1.2 ± 0.2 |
| CM15 | 49.3 ± 7.8 | 19.4 ± 2.7 | 17.5 ± 5.2 | 2.2 ± 0.6 | 4.1 ± 2.2 | 1.5 ± 0.1 | 0.5 ± 0.3 | 1.70 ± 0.83 | 0.5 ± 0.2 | 0.13 ± 0.16 | 3.3 ± 0.5 |
| CM16 | 61.8 ± 5.9 | 19.7 ± 1.9 | 7.6 ± 2.6 | 3.0 ± 1.3 | 3.0 ± 0.3 | 1.5 ± 0.1 | 0.6 ± 0.2 | 0.23 ± 0.06 | 0.4 ± 0.2 | 0.16 ± 0.07 | 2.0 ± 0.2 |
| CM17 | 48.6 ± 5.6 | 20.5 ± 2.9 | 13.7 ± 4.7 | 4.0 ± 0.8 | 4.3 ± 1.4 | 1.6 ± 0.3 | 1.1 ± 0.3 | 2.91 ± 0.68 | 0.6 ± 0.1 | 0.11 ± 0.07 | 2.6 ± 0.5 |
| CM18 | 44.5 ± 2.7 | 27.5 ± 5.5 | 4.4 ± 1.3 | 3.2 ± 1.3 | 7.9 ± 1.9 | 3.1 ± 0.6 | 2.7 ± 1.3 | 1.17 ± 0.56 | 1.7 ± 0.6 | 0.42 ± 0.18 | 3.5 ± 0.4 |
| Average | 61.1 ± 16.5 | 16.0 ± 8.0 | 8.6 ± 7.3 | 3.0 ± 3.2 | 3.5 ± 3.0 | 1.4 ± 1.0 | 0.7 ± 0.7 | 1.0 ± 3.0 | 0.5 ± 0.7 | 0.3 ± 0.7 | 3.8 ± 6.9 |

**TABLE S7** Relative abundance (%) of the dominant bacterial genera in rhizosphere soils of different species of *Caragana*.

| Sample | *Pseudomonas* | *Acinetobacter* | *Bacillus* | *Stenotrophomonas* | *Burkholderia* | *Paenibacillus* | *Sphingobacterium* | *Chitinophaga* | *Arthrobacter* | *Chryseobacterium* | Others |
| --- | --- | --- | --- | --- | --- | --- | --- | --- | --- | --- | --- |
| CI1 | 5.4 ± 0.7 | 0.8 ± 0.4 | 1.2 ± 0.2 | 0.7 ± 0.2 | 0.8 ± 0.2 | 0.7 ± 0.1 | 0.02 ± 0.00 | 0.04 ± 0.01 | 2.8 ± 2.0 | 0.02 ± 0.01 | 87.6 ± 3.7 |
| CI2 | 6.5 ± 1.9 | 0.8 ± 0.1 | 5.3 ± 1.1 | 1.0 ± 0.5 | 1.5 ± 0.5 | 0.9 ± 0.1 | 0.08 ± 0.06 | 0.50 ± 0.28 | 0.8 ± 0.1 | 0.03 ± 0.01 | 82.6 ± 3.5 |
| CI3 | 6.7 ± 1.7 | 0.7 ± 0.1 | 3.3 ± 0.9 | 2.7 ± 3.4 | 1.2 ± 0.6 | 0.9 ± 0.2 | 3.24 ± 5.53 | 0.25 ± 0.15 | 1.5 ± 0.3 | 0.04 ± 0.03 | 79.5 ± 9.1 |
| CI4 | 7.1 ± 0.7 | 1.3 ± 0.2 | 10.9 ± 1.5 | 0.2 ± 0.0 | 1.0 ± 0.2 | 0.9 ± 0.3 | 0.01 ± 0.01 | 0.72 ± 0.24 | 3.1 ± 0.6 | 0.03 ± 0.01 | 74.8 ± 1.2 |
| CI5 | 62.4 ± 8.1 | 1.1 ± 0.3 | 4.1 ± 1.0 | 0.1 ± 0.0 | 0.3 ± 0.2 | 0.2 ± 0.1 | 0.00 ± 0.00 | 0.15 ± 0.06 | 0.7 ± 0.2 | 0.00 ± 0.00 | 30.8 ±7.0 |
| CI6 | 43.4 ± 32.6 | 0.4 ± 0.2 | 2.9 ± 0.8 | 0.3 ± 0.3 | 0.3 ± 0.2 | 0.4 ± 0.3 | 0.03 ± 0.04 | 2.07 ± 3.37 | 1.8 ± 1.0 | 0.01 ± 0.00 | 48.4 ± 28.8 |
| CK1 | 2.0 ± 0.5 | 0.8 ± 0.7 | 2.9 ± 1.1 | 0.8 ± 0.6 | 0.3 ± 0.0 | 0.6 | 0.02 ± 0.02 | 0.54 ± 0.30 | 1.4 ± 0.2 | 0.04 ± 0.03 | 90.1 ± 1.4 |
| CK2 | 2.4 ± 0.8 | 2.1 ± 0.8 | 3.9 ± 2.0 | 0.1 ± 0.0 | 0.6 ± 0.2 | 0.9 ± 0.3 | 0.01 ± 0.01 | 0.46 ± 0.10 | 1.3 ± 0.3 | 0.02 ± 0.02 | 88.3 ± 3.1 |
| CK3 | 73.4 ± 4.2 | 0.2 ± 0.1 | 1.3 ± 0.6 | 0.1 ± 0.0 | 0.1 ± 0.0 | 0.3 ± 0.2 | 0.00 ± 0.01 | 0.09 ± 0.05 | 3.2 ± 0.5 | 0.03 ± 0.03 | 21.2 ± 3.6 |
| CK4 | 26.7 ± 42.6 | 0.4 ± 0.2 | 1.0 ± 0.3 | 0.1 ± 0.0 | 0.1 ± 0.0 | 0.5 ± 0.4 | 0.04 ± 0.03 | 2.65 ± 2.43 | 0.5 ± 0.3 | 0.04 ± 0.02 | 67.9 ± 39.5 |
| CK5 | 6.1 ± 0.1 | 1.6 ± 0.3 | 10.2 ± 0.3 | 0.1 ± 0.1 | 0.3 ± 0.4 | 1.7 ± 0.4 | 0.00 ± 0.00 | 0.22 ± 0.04 | 1.6 ± 0.5 | 0.00 ± 0.00 | 78.2 ± 0.8 |
| CM1 | 2.7 ± 0.9 | 1.6 ± 0.8 | 1.8 ± 0.6 | 1.1 ± 0.2 | 1.2 ± 0.1 | 0.7 ± 0.2 | 0.44 ± 0.12 | 0.24 ± 0.04 | 0.5 ± 0.1 | 0.31 ± 0.17 | 89.4 ± 0.7 |
| CM2 | 3.9 ± 1.8 | 1.3 ± 0.1 | 1.4 ± 0.8 | 0.3 ± 0.1 | 1.5 ± 1.7 | 0.7 ± 0.2 | 0.02 ± 0.00 | 0.09 ± 0.03 | 1.2 ± 0.7 | 0.00 ± 0.00 | 89.6 ± 4.8 |
| CM3 | 15.9 ± 1.6 | 4.0 ± 1.4 | 3.0 ± 0.8 | 0.3 ± 0.1 | 4.1 ± 3.0 | 1.0 ± 0.2 | 0.02 ± 0.01 | 0.18 ± 0.04 | 1.3 ± 0.1 | 0.06 ± 0.03 | 70.2 ± 3.1 |
| CM4 | 4.3 ± 4.6 | 1.0 ± 0.7 | 2.2 ± 1.3 | 10.5 ± 14.9 | 0.5 ± 0.1 | 5.5 ± 7.1 | 0.64 ± 0.94 | 0.18 ± 0.11 | 0.7 ± 0.2 | 0.14 ± 0.22 | 74.5 ± 17.5 |
| CM5 | 4.0 ± 3.1 | 1.9 ± 0.3 | 4.9 ± 2.6 | 0.2 ± 0.0 | 6.4 ± 9.2 | 3.5 ± 2.7 | 0.01 ± 0.00 | 0.06 ± 0.02 | 2.6 ± 1.8 | 0.00 ± 0.00 | 76.4 ± 7.9 |
| CM6 | 23.2 ± 4.7 | 3.0 ± 0.3 | 4.4 ± 1.4 | 0.4 ± 0.0 | 0.7 ± 0.1 | 0.3 ± 0.1 | 0.04 ± 0.00 | 0.11 ± 0.03 | 4.2 ± 1.3 | 0.04 ± 0.01 | 63.6 ± 3.0 |
| CM7 | 14.8 ± 5.0 | 2.4 ± 0.0 | 24.1 ± 4.3 | 0.5 ± 0.2 | 1.8 ± 0.1 | 0.4 ± 0.0 | 0.05 ± 0.01 | 2.36 ± 0.98 | 0.8 ± 0.2 | 0.08 ± 0.03 | 52.8 ± 9.9 |
| CM8 | 13.3 ± 9.4 | 2.7 ± 0.2 | 17.5 ± 9.4 | 0.7 ± 0.5 | 3.2 ± 2.0 | 0.5 ± 0.1 | 0.16 ± 0.12 | 0.72 ± 0.51 | 1.7 ± 0.3 | 0.11 ± 0.02 | 59.4 ± 4.5 |
| CM9 | 6.8 ± 2.0 | 6.7 ± 4.2 | 3.3 ± 0.8 | 1.7 ± 0.3 | 1.7 ± 0.4 | 0.2 ± 0.0 | 8.76 ± 3.2 | 0.17 ± 0.01 | 0.5 ± 0.1 | 3.50 ± 2.09 | 66.7 ± 9.9 |
| CM10 | 14.1 ± 12.9 | 16.0 ± 12.0 | 3.9 ± 1.1 | 0.3 ± 0.2 | 0.9 ± 0.7 | 0.4 ± 0.1 | 0.07 ± 0.05 | 0.11 ± 0.06 | 3.1 ± 1.1 | 0.05 ± 0.01 | 61.1 ± 9.4 |
| CM11 | 22.8 ± 6.4 | 5.5 ± 0.5 | 9.7 ± 1.6 | 0.3 ± 0.1 | 3.6 ± 1.9 | 1.2 ± 0.3 | 0.07 ± 0.06 | 0.55 ± 0.32 | 5.0 ± 0.8 | 0.06 ± 0.03 | 51.1 ± 5.6 |
| CM12 | 4.5 ± 0.5 | 14.8 ± 22.2 | 11.1 ± 8.0 | 1.1 ± 0.8 | 0.5 ± 0.1 | 1.4 ± 0.7 | 0.07 ± 0.05 | 0.22 ± 0.15 | 1.6 ± 0.6 | 0.08 ± 0.02 | 64.5 ± 17.6 |
| CM13 | 8.9 ± 2.6 | 34.6 ± 11.3 | 13.7 ± 13.7 | 0.1 ± 0.0 | 1.1 ± 0.6 | 0.8 ± 0.5 | 0.03 ± 0.01 | 0.10 ± 0.05 | 0.7 ± 0.0 | 0.03 ± 0.01 | 39.8 ± 2.7 |
| CM14 | 8.4 ± 0.9 | 0.6 ± 0.3 | 1.7 ± 0.2 | 0.5 ± 0.1 | 0.6 ± 0.1 | 0.2 ± 0.0 | 0.02 ± 0.01 | 0.10 ± 0.02 | 0.9 ± 0.2 | 0.01 ± 0.01 | 87.1 ± 1.0 |
| CM15 | 12.6 ± 10.8 | 0.6 ± 0.1 | 16.1 ± 5.2 | 0.4 ± 0.2 | 1.9 ± 1.8 | 0.5 ± 0.2 | 0.05 ± 0.08 | 0.56 ± 0.47 | 1.8 ± 0.8 | 0.02 ± 0.02 | 65.4 ± 6.6 |
| CM16 | 19.6 ± 12.7 | 1.2 ± 0.4 | 6.5 ± 2.3 | 0.6 ± 0.4 | 0.5 ± 0.4 | 0.3 ± 0.1 | 0.06 ± 0.06 | 0.54 ± 0.27 | 4.5 ± 0.9 | 0.08 ± 0.05 | 66.2 ± 9.2 |
| CM17 | 3.6 ± 1.3 | 1.3 ± 0.7 | 12.7 ± 4.6 | 0.6 ± 0.3 | 1.2 ± 0.3 | 0.4 ± 0.1 | 0.15 ± 0.09 | 0.86 ± 0.38 | 1.4 ± 0.4 | 0.20 ± 0.15 | 77.6 ± 3.0 |
| CM18 | 2.8 ± 0.6 | 1.3 ± 0.2 | 2.8 ± 0.9 | 0.2 ± 0.1 | 0.6 ± 0.3 | 0.5 ± 0.1 | 0.00 ± 0.01 | 0.39 ± 0.07 | 1.4 ± 0.1 | 0.00 ± 0.00 | 90.1 ± 1.5 |
| Average | 14.9 ± 19.6 | 3.8 ± 8.2 | 6.4 ± 6.6 | 0.9 ± 3.0 | 1.3 ± 2.1 | 0.9 ± 1.6 | 0.5 ± 2.0 | 0.5 ± 1.0 | 1.8 ± 1.4 | 0.2 ± 0.7 | 68.7 ± 20.3 |

**TABLE S8** The best combinations of environmental factors and soil properties explained the bacterial community variation.

| Combinations | % variance explained | *p* value |
| --- | --- | --- |
| TP | 9.7 | 0.002 |
| TP+TN | 16.6 | 0.002 |
| TP+TN+MAT | 21.9 | 0.002 |
| TP+TN+MAT+RH | 29.6 | 0.002 |
| TP+TN+MAT+RH+EC | 34.2 | 0.002 |
| TP+TN+MAT+RH+EC+MAP | 38.7 | 0.002 |
| TP+TN+MAT+RH+EC+MAP+PM | 41.8 | 0.002 |
| TP+TN+MAT+RH+EC+MAP+PM+TC | 44.1 | 0.002 |
| TP+TN+MAT+RH+EC+MAP+PM+TC+pH | 46.6 | 0.002 |
| TP+TN+MAT+RH+EC+MAP+PM+TC+pH+Alt | 47.5 | 0.002 |

Notes: MAP, mean annual precipitation; MAT, mean annual temperature; RH, relative humidity; PM, Penman-Montieth; Alt, altitude; TP, total phosphorus content; TN, total nitrogen content; TOC, total organic carbon; EC, electrical conductivity.
